# Study protocol for prevention of falls: A randomized controlled trial of effects of vitamin D and exercise on falls prevention

**DOI:** 10.1186/1471-2318-12-12

**Published:** 2012-03-26

**Authors:** Kirsti Uusi-Rasi, Pekka Kannus, Saija Karinkanta, Matti Pasanen, Radhika Patil, Christel Lamberg-Allardt, Harri Sievänen

**Affiliations:** 1 The UKK Institute for Health Promotion Research, PO Box 30, FI-33501 Tampere, Finland; 2 Pirkanmaa Hospital District, Science Center, PO Box 2000, FI-33521 Tampere, Finland; 3 Medical School, University of Tampere, 33014 Tampere, Finland; 4 Division of Orthopaedics and Traumatology, Department of Trauma, Musculoskeletal Surgery and Rehabilitation, Tampere University Hospital, PO Box 2000, FI-33521 Tampere, Finland; 5 Department of Food and Environmental Sciences, University of Helsinki, PO Box 66, FI-00014 Helsinki, Finland

**Keywords:** Exercise, Falls, Physical functioning, Vitamin D, Mobility function, Neuromuscular functioning, Quality of life

## Abstract

**Background:**

Falls are the leading cause of unintentional injury and injury-related death among older people. In addition to physical activity, vitamin D also may affect balance and neuromuscular function. Low serum 25-hydroksivitamin D level increases the risk of bone loss, falls and fractures. Thus, an appropriate exercise program and sufficient vitamin D intake may significantly improve not only functional balance, but also balance confidence. Balance represents a complex motor skill determined by reaction time, muscle strength, and speed and coordination of movement.

**Methods/Design:**

A 2-year randomized double-blind placebo-controlled vitamin D and open exercise trial of 409 home-dwelling women 70 to 80 years of age comprising four study arms: 1) exercise + vitamin D (800 IU/d), 2) exercise + placebo, 3) no exercise + vitamin D (800 IU/d), 4) no exercise + placebo. In addition to monthly fall diaries, general health status, life style, bone health, physical functioning, and vitamin D metabolism will be assessed. The primary outcomes are the rate of falls and fall-related injuries. Secondary outcomes include changes in neuromuscular functioning (e.g. body balance, muscle strength), ADL- and mobility functions, bone density and structure, cardiovascular risk factors, quality of life and fear of falling.

**Discussion:**

The successful completion of this trial will provide evidence on the effectiveness of exercise and vitamin D for falls reduction.

**Trial Registration:**

ClinicalTrial.gov -register (NCT00986466).

## Background

The rising population of the elderly over the next few decades will be accompanied by an increase in the number of people with disease and chronic illness. Older people are less resistant to injury, whether from physiological events (e.g. heart attack) or environmental trauma (e.g. bone fracture), as well as to infection [1]. Health maintenance for aging people over their life span through exercise and proper nutrition contribute to lifelong wellbeing [2].

Osteoporosis, with the main outcome problem of fractures, is a multifactorial disease characterized by low bone mineral density (BMD) and decreased bone strength. Often these people also have neuromuscular deficiencies resulting in an increased risk of falling. Falls are the leading cause of unintentional injury and injury-related death among older adults. Approximately 30% of community living people aged 65 years or older fall every year the rate being clearly higher in institutions. Although less than one fall out of 10 results in a fracture, 20% of fall incidents require admissions to hospital [3-5]. Since the propensity to fall increases the risk of fracture and other injuries considerably, falls prevention is widely considered to be the most essential element when planning effective injury and fracture prevention programs for the elderly population [3,6-8].

Preventing falls and injuries among older adults is challenging. However, there is strong high-quality evidence from randomized controlled trials and subsequent systematic reviews and meta-analyses that regular strength and balance training for elderly adults living in the community can reduce the risk of both noninjurious and injurious falls by 15-50% [3,7,9,10]. Randomized controlled trials indicate that not only individually tailored training but also untargeted group exercise programs are effective in preventing falls [11-14], particularly if the training program includes Tai Chi or other exercises which challenge balance [14-16] and improve lower limb muscle strength [8,10]. Thus, it seems prudent to recommend regular weight-bearing and other exercises for community-dwelling older adults, not only to maintain their musculoskeletal health, but also to help them stay safely on their feet [17-19].

Hypovitaminosis D is becoming a widespread concern around the world regardless of the latitude. Serum 25-hydroxy vitamin D (S-25-OHD) concentration, which is the best indictor of vitamin D status, has been shown to decrease in elderly Finnish women in winter [20,21]. It has been estimated that ambulatory elderly women require on average 18 μg vitamin D per day to maintain adequate vitamin D level (that is, S-25-OHD above 50 nmol/L) during winter. Inadequate intakes of vitamin D and calcium lead to reduced calcium absorption, higher bone turnover and increased bone loss and risk of fractures [22-25]. Even more important may be the finding that S-25-OHD level is inversely associated with falls [24,26-28]. In randomized controlled trials the incidence of falls was almost halved and musculoskeletal function improved among elderly people with a combination of vitamin D and calcium compared with calcium supplement alone [29,30]. Falling may, at least partly, be a consequence of impaired neuromuscular function associated with vitamin D deficiency, since abnormal motor performance, increased body sway, and quadriceps weakness have been reported in those with low vitamin D status. Vitamin D receptors are known to be present in muscle tissue. Furthermore, the number of fast type II muscle fibers decreases with age, and these fibers are the first to be recruited in balance perturbation to avoid falling [31,32].

In prevention of fractures of elderly people, the focus should be shifted from treating low BMD to improving muscle strength and the neuromuscular co-ordination to avoid falls, since most fractures are a direct consequence of falling [33]. Although a recently published metaanalysis found little evidence that current multifactorial falls prevention programs could prevent falls and related injuries [34], it must be kept in mind that a great majority of fall-prone elderly adults has more than one risk factor for falls. Furthermore, fear of falling may restrict habitual physical activity and lead to declined functional ability [19,35,36].

As noted above, randomized controlled trials suggest that exercise may effectively improve many factors affecting falling, such as muscle strength, flexibility, balance, coordination, proprioception, reaction time and gait [37-41]; thus reducing the risk of falling [3,7-9,42]. Regular moderate to vigorous exercise is also associated with reduced number of fractures [18,43,44]. Also vitamin D has been proposed to protect against falls and fractures [27]. Thus, it is well justified to study the separate and combined effects of exercise and vitamin D in prevention of falls and related injuries.

Although there is evidence that both exercise and vitamin D by themselves can benefit neuromuscular and cognitive function, and thus contribute to reduced risk of falls; these two factors have not previously been evaluated in combination. In this paper, we describe the design of such a study.

## Methods/Design

### Study aims, design and setting

The present study is a four-arm randomized, double-blind placebo-controlled vitamin D and open exercise intervention trial where community-dwelling, independently living Finnish women aged 70 to 80 years are randomized to receive either vitamin D 800 IU (20 μg/d) or placebo, and to participate in either supervised exercise or non-exercise group for 24 months (DEX study).

The primary aim of this study is to investigate the effects of exercise and vitamin D supplementation, alone or in combination, on reducing falls and fall-related injuries.

### Ethical consideration

The study protocol has been approved by the Ethics Committee of the Tampere University Hospital, Tampere, Finland (R09090). Each participant provided her written informed consent prior to randomization. The study protocol is registered in the ClinicalTrial.gov - register (NCT00986466).

### Outcomes

The primary outcome of the study is the rate of falls and fall-related injuries. Changes in neuromuscular functioning (e.g. body balance, muscle strength), ADL- and mobility functions, bone health, fractures, cardiovascular risk factors, health-related quality of life, fear of falling, depression (WHO-5), cognition (Mini-mental State examination, MMSE) and rate of institutionalization will be analyzed as the secondary outcomes.

### Sample size consideration

The sample size and power calculations have been estimated for the primary outcome of this study, i.e., the rate of falls. We hypothesized that there will be a 30% difference in the rate of falls between the treatment groups (vitamin D vs. placebo, and exercise vs. non-exercise). The number of women in each group is based on the expectation that the intervention programme (exercise or vitamin D treatment) will reduce the incidence rate of falls by 30% during the two years intervention and the between-group difference will be detected for a given significance level of 0.05 with a statistical power of 80% with a mean treatment time of 1.5 years. The baseline incidence was assumed to be 67 falls per 100 person-years. Accordingly 260 participants needed to be recruited into the study (130 exercisers and 130 non-exercisers, 65 in each group with vitamin D supplement). However, in order to eliminate the role of chance in detecting the possible interaction of vitamin D and exercise, a total number of 400 subjects (100 subjects in each group) were recruited into the study.

### Screening for inclusion

**Figure 1:**
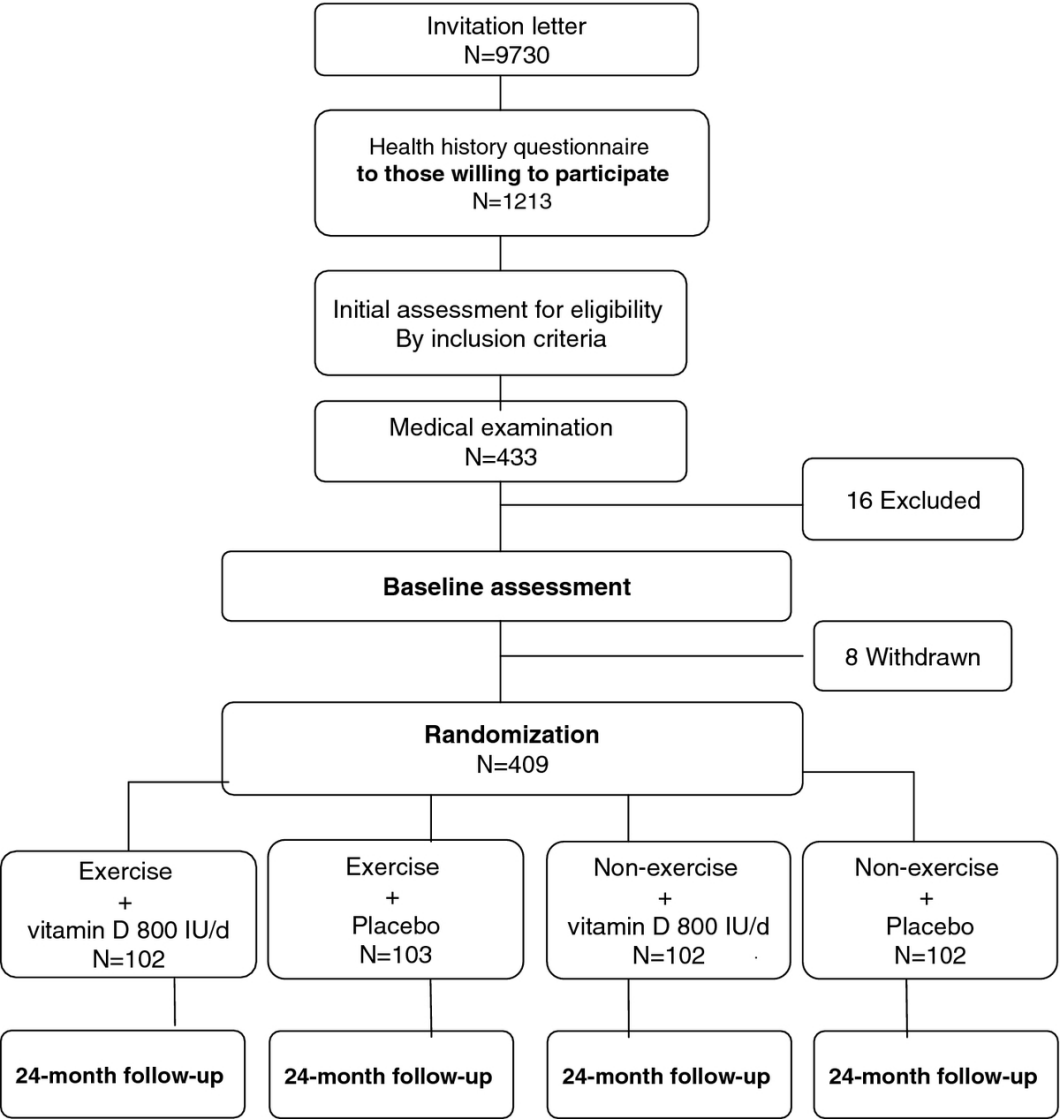
**Flowchart of the randomized control trial**.

Flowchart of the trial is shown in Figure 1. All 70 to 80-year old women living in the city of Tampere, Finland (n = 9370) were invited to participate in the trial. In addition to willingness, history of at least one fall during the last 12 months and no regular use of vitamin D supplements were the two other primary criteria mentioned in the first contact letter.

A pre-screening *Health history questionnaire *(information of self-reported health, previous falls, injuries, medication, diseases, and life style factors such as diet, physical activity, smoking and consumption of alcohol) was mailed to 1213 (12.9%) responders who expressed their initial interest and 433 women initially considered eligible for the study were then invited to the medical examination. The inclusion criteria were age between 70-80 years, living at home independently; had fallen at least once during the previous year; no contraindication to exercise; understands the procedures of the study, has been informed of X-ray radiation doses of the DXA and pQCT investigations, and amount of blood samples needed, and voluntarily agrees to undergo all measurements and signs informed consent prior to beginning of the procedure. The exclusion criteria were moderate to vigorous exercise more than 2 hours per week; regular use of vitamin D or calcium + vitamin D supplements; a recent fracture (during preceding 12 months); contraindication or inability to participate in the exercise program; a marked decline in the basic activities of daily living (ADL); cognitive impairments (Mini Mental State Examination, MMSE-test); primary hyperthyroidism; and degenerative conditions, such as Parkinson's disease.

A physician excluded 16 additional women due to exclusion criteria, and 8 withdrew after the baseline measurements leaving 409 eligible women for randomization.

### Randomization

409 participants were randomly assigned into one of the four arms using a computer-generated randomization list (based on simple randomization with random allocation sequence to ensure equal group sizes). The four arms are: 1) exercise + vitamin D (800 IU/d) 2) exercise + placebo 3) no exercise + vitamin D (800 IU/d) 4) no exercise + placebo.

### Participant follow-up procedures

#### Falls

The number of falls will be monitored with daily fall diaries. Diaries will be collected monthly through the mail. Details of each registered fall will be ascertained by the investigator. The definition of a fall is "an unexpected event in which the participant come to rest on the ground, floor or lower level" [45]. Injurious falls are those requiring medical attention and treatment. Minor injuries, such as bruises, edemas, muscular or joint pain, will also be recorded.

### Measurements

All measurements will be done at baseline, at 12-, and 24-month (the end point of the intervention). Blood samples and physical functioning will be assessed also at 6, and 18-month time points.

#### General health status and falls consequences

Information on the participant's health, medication, lifestyle (level of physical activity, use of alcohol, smoking), mobility and cognitive functions will be assessed with appropriate methods. The methods to evaluate functional ability, health-related quality of life (Leipad, Finnish version), fear of falling (Falls Efficacy Scale-International, FES-I, Finnish version) and physical activity (CHAMPS -activities questionnaire for older people) are modified suitable for the Finnish culture.

#### Anthropometry

Body height will be measured to the nearest 0.1 cm, and body weight to the nearest 0.1 kg with a high-precision scale. Body composition (fat mass and lean body mass) will be assessed with dual-energy X-ray absorptiometry (DXA, Lunar Prodigy Advance, GE Lunar, Madison, WI, USA). According to repeated measurements of 22 adults, the *in vivo *precision (coefficient of variation, CV%) was 1.3% for fat mass and 0.7% for fat-free lean mass (Sievänen, unpublished).

#### Bone measurements

Bone mineral content (BMC) of the total body, lumbar spine and left proximal femur (femoral neck and trochanter) will be assessed using dual-energy X-ray absorptiometry (DXA) (Lunar Prodigy Advanced, GE Lunar, Madison, WI, USA). At our laboratory, the in vivo day-to-day precision (coefficient of variation, CV%) of the DXA scanning is better than 1% for the lumbar spine and 1.5% for the proximal femur (Sievänen 2005, unpublished). In addition to bone mass, strength of the femoral neck will be estimated from the DXA-measurements using the AHA-software.

In addition to the DXA measurements, the tibia will be measured with peripheral quantitative computed tomography (pQCT) (Norland/Stratec XCT 3000, Pforzheim, Germany). The tomographic slices will be taken from the midshaft (50%) and distal part (5% of the tibial length from the distal end plate of the tibia) for assessing bone mineral mass, cortical and trabecular apparent densities as well as strength traits of this weight-bearing bone [46].

#### Physical performance, physical functioning, and physical activity

The maximal isometric leg-extensor strength at the knee ankle 110° will be measured by a strain gauge dynamometer (Tamtron, Tampere, Finland) and the grip strength of both forearms with a standard hand dynamometer (Lafaytte, Loughborough, UK). The participants are verbally encouraged to perform to their maximum and the best performance from three trials will be taken as a test result. Timed up and go test (TUG) [47], Short Physical Performance Battery (SPPB) [48,49] and backwards walking [50,51] will be used for assessing physical functioning. In addition to CHAMPS, each participant's daily number of steps will be measured with a pedometer (Omron HJ-112-E) over the entire 24-month study period.

#### Vitamin D status

Dietary intake of calcium and vitamin D will be assessed with a validated food frequency questionnaire [52] and calculated by Micro-Nutrica software (Social Insurance Institution, Helsinki, Finland). Serum 25-hydroxy-vitamin D (S-25-OHD) will be measured as a marker of vitamin D-metabolism with an OCTEIA immunoenzymometric assay (IDS, Bolton, UK). In addition, serum intact parathyroid hormone (S-iPTH) will be measured (OCTEIA, IDS), as it usually increases when S-25OHD is low, and gives additional information on the severity of vitamin D deficiency. Genomic DNA sample will be taken to find possible gene polymorphism associated with vitamin D metabolism (e.g. the vitamin D receptor, VDR).

#### Plan of analysis

The incidence rate will be calculated as the number of falls divided by the time over which falls are monitored for each participant. The data will be analyzed as a 2 × 2 factorial design and Poisson regression models will be used to estimate incidence rate ratio between the two exercise groups and between the two vitamin D groups for falls and injuries falls. Between-group changes in neuromuscular functioning, physical ability, bone traits, and quality of life will be analyzed by analysis of covariance (ANCOVA) or logistic regression analysis.

### Intervention

#### Vitamin D supplements/placebo

The participants were randomly assigned to receive placebo (50% of the participants) or 800 IU (20 μg) of vitamin D (50% of the participants) per day for two years. Both participants and outcome assessors are blinded to the group assignment of placebo or vitamin D during the study. At the start, each participant received a pack of pills for six months, and when arriving to the follow-up measurements at six-month intervals the used packs will be returned and new full packs will be given. At this time, compliance will be confirmed by remaining pill counts. A questionnaire on side effects will be administered to all participants at six-month intervals to monitor safety. As standard safety markers, S-Ca and S-Pi will be assayed.

### Training program

Participants randomized to the exercise groups (50% of the participants either on vitamin D or placebo) will attend supervised training classes 2 times a week for the first 12 months, and once a week for the last 12 months of the 24-month intervention. In addition, they receive a home exercise plan to be practised on the rest days.

All group training sessions are supervised by 1 or 2 experienced exercise leaders (physiotherapists). The training program is progressive and consists of strength, balance, agility and mobility training. Main components of the exercise program has been found safe and feasible in the present age group in our previous study [38]. Training sessions are carried out in 8-week periods, either in the exercise hall or gym. Around 10 to 20 participants are expected to attend these training sessions. All training sessions last 60 minutes and include a 10 minutes warm-up as well as stretching for major muscle groups. A 4-week familiarizing period precedes the first 8-week training period to accustom the exercisers to the training, and to familiarize them to each other and the exercise leaders.

During the group exercises in hall, exercises focusing on balance, agility, mobility and change of direction are emphasized. Different surfaces and dual-task situations are employed, especially in balance and agility exercises. In addition to own body weight, ankle weights and vest weights are used in muscle strength exercises. Some exercises will be done with a chair (especially at the beginning of the training) or with a step-board (when advanced). The advanced programs are also aerobic in nature. Exercise programs will either be led by the leaders or implemented as supervised circuit training. Exercise leaders keep a record of the participants' attendance at the group training sessions.

During the gym exercises, resistive equipment is used. All programs include 8-9 different exercises focusing on strengthening large muscle groups: leg extensors (leg press), hip extensors, hip abductors, knee extensors, knee flexors, calf muscles, back muscles, and shoulder and arm muscles. The first gym period begins with 30 - 60% of one repetition maximum (1RM). The target level progresses to 60-75% of 1RM. Two sets of each exercise are done, each set consisting of 8-12 repetitions. The intensity of strength training is assessed using rated perceived exertion (RPE) [53]. The target RPE ranges from 14-18 (12 when starting), and advances progressively. Balance training is included in a short warm-up period.

In addition to supervised training sessions, the exercisers have a home-training program modified from the exercises used during training sessions. During the first year the participants are guided to train at home in the days they do not participate in the supervised group training. In the second year, home-training sessions are recommended at least 3 times a week. One home training session requires 5 to 15 minutes. The exercisers keep a home training diary.

The participants in the non-exercising group (50% of the participants either on vitamin D or placebo) are asked to maintain their current level of physical activity.

### Time schedule

Preparations for recruitment of the participants were completed during the spring 2009. The screening and baseline measurements of the participants began in fall 2009. For practical reasons, the trial started in two phases (201 participants in March 2010 and 208 in March 2011), the follow-up measurements will be carried out in spring 2012 and 2013, respectively.

## Discussion

At the time of writing, recruitment was completed and all participants had been randomized and respective interventions started as planned. The 24-month measurements and the follow-up of the first group will be completed in March 2012, and the second group in March 2013. Results will be available late 2013. Although it is not possible to comment on the effectiveness of the intervention, it is evident that the participants are well committed to the study.

## Competing interests

The authors declare that they have no competing interests.

## Authors' contributions

KUR developed the idea for the study and obtained funding. PK, SK, MP, HS and CLA were involved in elaborating specific research questions and designing the trial protocol. PK, SK, MP and HS also contributed to methodological decisions such as study sample and choice of outcome measures. SK and RP were responsible for planning of the training program. CLA provides the methodological support for biochemical laboratory assays. KUR, SK, PK, MP, RP and HS are responsible for data collection and management and MP for statistical analyses of the trial data. KUR drafted the manuscript. All authors commented on the draft and read and approved the final version of the paper.

## Pre-publication history

The pre-publication history for this paper can be accessed here:

http://www.biomedcentral.com/1471-2318/12/12/prepub
